# A discrepancy of 10^7^ in experimental and theoretical density detection limits of aerosol particles by surface nonlinear light scattering

**DOI:** 10.1038/s42004-023-00903-8

**Published:** 2023-06-08

**Authors:** Arianna Marchioro, Thaddeus W. Golbek, Adam S. Chatterley, Tobias Weidner, Sylvie Roke

**Affiliations:** 1 Laboratory for Fundamental BioPhotonics (LBP), Institute of Bioengineering (IBI), and Institute of Materials Science (IMX), School of Engineering (STI), and Lausanne Centre for Ultrafast Science (LACUS), Ecole Polytechnique Fédérale de Lausanne (EPFL), Lausanne, Switzerland; 2 Department of Chemistry, Aarhus University, Aarhus C, Denmark

**Keywords:** Optical spectroscopy, Surface spectroscopy, Nanoparticles, Atmospheric chemistry

**arising from** Y. Qian et al. *Communications Chemistry* 10.1038/s42004-022-00674-8 (2022)

Aerosol science would benefit greatly from understanding the interfacial structure of airborne particles. However, measuring the surface structure in situ is challenging because of the weak second-order nonlinearity needed to generate a surface response, the dispersive/scattering nature of the sample, the dilution of particles (~10^6^/mL), and the size range of the majority of them (10–300 nm, in diameter)^1^. Indeed, vibrational surface SFS spectra, as well as non-resonant surface second harmonic scattering (SHS, Fig. 1a, which is comparable) measured from particles dispersed in solution of this size range, have been reported with samples containing ~10^11^ particles/mL^2,3^. There is no principle difference in the mechanism and rules that govern nonlinear scattering for particles that are airborne or dispersed in solution^4^, with the difference in surrounding medium being incorporated by the difference in refractive index between the particle and surrounding medium. Since the 1980’s numerous groups have worked on developing and verifying nonlinear light scattering theories, and these theories, like their linear counterpart, generally agree with nonlinear light scattering experiments^5^. Indeed, the single difference between linear and nonlinear light scattering is the induced polarization of the material that is used as a source term. Therefore, the results of the Article this contribution is arising from^1^ raise questions as to the nature of the source of the SF scattering object(s), as there is a difference on the order of ~10^7^ between the expected scattering power and the detected spectra.

**Fig. 1:**
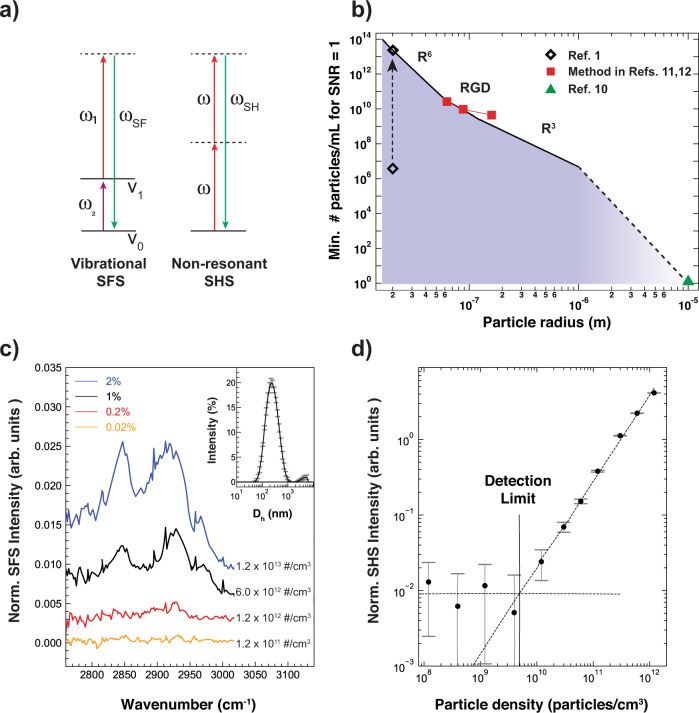
Number density vs size dependence. **a** Illustration of energy schemes of vibrational sum frequency scattering and non-resonant second harmonic scattering. **b** Number density necessary to generate a signal-to-noise ratio = 1 as a function of particle size, assuming the minimal detectable intensity comprises 10 % of the non-resonant SHS response of neat bulk water. Black diamonds, red squares and green triangle correspond to data points extracted from the indicated references. The colored area is inaccessible to current non-resonant SHS and vibrational SFS instruments. **c**, **d** Vibrational SFS **c** and non-resonant SHS measurements **d** of SDS stabilized d_34_-hexadecane nanodroplets in D_2_O measured at different droplet densities, ranging from 1.2 × 10^13^/cm^3^ (blue trace) to 1.2 × 10^11^/cm^3^ (orange trace, which we consider the detection limit). The number of droplets is computed from the used volume in combination with the hydrodynamic diameter (D_h_) measured by DLS (shown in the inset of **c**). The spectra in the SFS plot are offset for clarity. The SHS measured intensity (**d** black dots) and standard error (grey error bars) are plotted with trend lines for both the intensity above and below the noise level (dashed lines). The detection limit was determined to be at the intersection of both lines at 4.9 × 10^9^/cm^3^.

Here, we first explicitly determine the number of particles that can be reasonably measured for a certain size in SHS/SFS experiments. To do so, we combine theoretical predictions per particle with experiment. We will first consider the throughput of the SFS/SHS experiment and consider its size and number density dependence, then provide a signal-to-noise ratio analysis of various comparable experiments. Using this analysis in combination with SFS and SHS experiments performed on the same samples, we determine the detection limit in terms of particle density for a certain size. Finally, we discuss several explanations for the difference in terms of detected intensity and expected aerosol density.

## Size dependence

Vibrational SFS and non-resonant SHS are second-order nonlinear optical techniques. The intensity of the generated photons obeys the following expression:

1 $$I({\omega }_{0})\propto {N}_{p}{\left|{\Gamma }^{\left(2\right)}(R,\theta ,{\chi }^{\left(2\right)})\right|}^{2}\frac{{E}_{1}{E}_{2}}{\tau A}f$$

with *N*_*p*_ the particle density, ${\Gamma }^{\left(2\right)}(R,\,\theta ,{\chi }^{\left(2\right)})$ the effective (single) particle susceptibility, which depends on the radius *R*, the scattering angle *θ*, the (surface) susceptibility of the particle *χ*^(2)^, *E*_*i*_ the pulse energies of the participating beams, *τ* the pulse duration, *A* the overlap area and *f* the repetition rate. The effective particle susceptibility that determines the response of a single particle ${\Gamma }^{\left(2\right)}(R,\,\theta ,{\chi }^{\left(2\right)})$ is determined by the single particle light-matter interaction process and is highly size dependent. ${\Gamma }^{\left(2\right)}$ also contains effects of absorption/linear scattering as it is also a function of the electromagnetic field functions^6^. For *R*~<200 nm, ${\Gamma }^{\left(2\right)}\propto \,{R}^{3}$ $(I({\omega }_{0})\propto {R}^{6})$, and for larger particles, this size dependence levels off, reaching $I({\omega }_{0})\propto {R}^{3}$ at *R*~1000 nm. For *R* <200 nm, the Rayleigh-Gans-Debye approximation, one of the approximate solutions to the Maxwell equations, works well to describe the data. For larger sizes, it becomes more approximate, and depending on the conditions nonlinear Mie theory needs to be used^4^. Nonlinear Mie theory offers an exact solution for both linear and nonlinear scattering, assuming the single scattering particles are spherical. Dipolar and quadrupolar scattering both lead to identical size-dependent behaviors^4,7^. The scattering pattern is also highly size dependent with scattering maxima appearing between 90° and 55° for *R* < 50 nm, which gradually move forward. For water droplets in the air, however, the refractive index contrast ensures that for micron-sized or larger particles scattering light is emitted in every direction^4^. Particles in the air have a bigger linear refractive index difference between the bulk and the particle medium compared to solid/liquid dispersions, which generally have a smaller refractive index contrast and therefore exhibit less losses due to linear scattering of the incident beams. We can expect that the estimations based on the systems used in this work are over-estimating the actual scattering efficiencies.

## Signal-to-noise ratio

To relate the experimental throughput of the experiments presented in the Article this contribution is arising from^1^, we first compare their signal-to-noise ratio (SNR) to previously published vibrational SFS and non-resonant SHS data, and then perform vibrational SFS and non-resonant SHS measurements on hexadecane nanodroplets. Table 1 shows the experimental parameters that relate the 3 published experiments, as well as the parameters used to collect the data in Fig. 1c, d. The SFS experiment reported in the Article this contribution is arising from^1^ has a comparable SNR compared to previously published SFS data and a 1.4–2.4 smaller SNR to non-resonant SHS. Because SHS and SFS have comparable SNRs, we retrieve the particle vs size dependence from SHS. The primary reason to do so is that the SHS intensity of any particle surface can be compared both experimentally and theoretically to the known incoherent bulk response of neat water, which therefore represents a calibration benchmark. This incoherent SH light is known as hyper Rayleigh scattering, and is subtracted from the raw data, to obtain the pure particle scattering (Eq. S1). With a known hyperpolarizability tensor of water, it is therefore possible to explicitly compute the theoretical response that matches 10% of the magnitude of the intensity recorded with SNR = 1. We take this as the detection limit of the SFS and non-resonant SHS experiments of particles. Thus, we compute using the theory in Refs. ^8,9^, as a function of particle radius, which number density of particles is needed to generate an intensity that matches 10% of the incoherent neat bulk water intensity in the SSS polarization combination (all beams polarized perpendicular to the scattering plane). We insert typical values for the surface susceptibility (*χ*^(2)^ = 10^−22^ m^2^/V) and the surface potential (*Φ*_0_ = 100 mV), which represent the surface properties of the particles.

**Table 1:** Experimental parameters.

	SFS as in Ref. ^1^	SFS as in Refs. ^2,13^	SHS as in Refs. ^11,12^	SFS, Fig. 1c	SHS Fig. 1d
Fundamental wavelength	1025 nm	800 nm	1030 nm	517 nm	1032 nm
Repetition rate	100 kHz	1 kHz	200 kHz	10 kHz	200 kHz
OPA range	2500–4500 nm	2600–20000 nm	NA	2300–15000 nm	NA
IR pulse energy at the sample (μJ)	2	5-10	0.3	13.6	0.4
Beam waist (diameter, μm)	80	340	110	400	110
Fluence (mJ/cm^2^)	39.8	5.5	3.2	10.8	4.2
VIS pulse linewidth (cm^−1^)	8 cm^−1^	12 cm^−1^	NA	0.18 nm	NA
VIS pulse energy (μJ)	6	5–10	NA	7.5	NA
Collection angle range (θ)	90° ± 30°	θ_max_ ± 10°	−90° < θ < +90° (3.4°/θ)	θ_max_ ± 10°	−90° < θ < +90° (approx. 4°/θ)
Max. SNR ratio (PPP or SSP)	11	5–15	16–26	36	15
SNR ratio SSS bulk H_2_O			18–21		~15

PPP (SSS) refers to all beams polarized in (perpendicular to) the scattering plane.

## Estimation of particle density vs size dependence

The line in Fig. 1b shows the detection limit in terms of # particles/mL as a function of the radius of the particles, based on extrapolating the various size-dependent models^4^. For a 20 nm radius, this means 2.5 × 10^13^ particles/mL are needed to generate the desired SF intensity. Moving to larger particles, we arrive at 5.6 × 10^10^ particles/mL (50 nm radius), 4.2 × 10^7^ (500 nm radius), and finally, extrapolating to non-resonant SH imaging, 1 for objects in the size range of 5–10 microns^10^. For diameters close to 100, 200, and 300 nm, data points from angle-resolved non-resonant second harmonic scattering measurements from the surface of silica particles dispersed in water^11,12^ are shown. Based on this result, we estimate that 10^6^ particles/mL can only generate a detectable response if R > 1000 nm. Alternatively, 40 nm particles would have to be present at a density of 2.5 × 10^13^ particles/mL, as indicated by the dashed arrow. Neither of these criteria are met in the Article this contribution is arising from^1^.

Fig. 1c shows SFS spectra of deuterated hexadecane droplets with a 109 ± 1 nm average hydrodynamic radius stabilized with 8 mM sodium dodecyl sulfate (SDS), following the protocol of Chen et al.^13^ and summarized in the Supplementary Methods. SFS spectra were recorded with particle densities between 10^11^/mL and 10^13^/mL, achieved by diluting a stock emulsion (size distribution is shown in the inset). In agreement with Fig. 1b, the recorded SF intensity vanishes below a particle density of 10^12^/mL. Figure 1d shows data measured by non-resonant SHS of the same sample diluted with 0.8 mM SDS solution. In both experiments, no detectable signal can be obtained below 4.9 × 10^9^ particles/mL, in agreement with predictions of Fig. 1b for identically-sized objects.

## Discussion and conclusions

Although the values of *χ*^(2)^, the scattering angle range, the precise value of the refractive indices, or the surface potential may change, given the difference examined here (a factor of 10^7^ for a particle of 40 nm in diameter) these details are relatively unimportant as they would create a maximum difference in the scattering intensity in the range 0.1–10. Since the instrument used in the Article this contribution is arising from^1^ is not outperforming the instruments used here (Table 1), this brings about the question what actually generates the unexpectedly strong SFS response. Based on nonlinear light scattering theory summarized by Eq. (1), there are only two candidates for increasing the scattered intensity by ×10^7^: The size of the particles, with the scattering objects being much larger than reported, or *χ*^(2)^, which should then increase by ~10^3^. A single particle of a few microns in size could easily overpower the emission of all other particles and generate the measured SF intensity, for example $\frac{I(1\,\mu m)}{I(10\,{nm})} \sim ({10}^{2})^{3}$. To get insight here, one would have to measure the SF scattering patterns, as was done in Ref. ^2^. A time-dependent intensity trace would provide information about the stability of the intensity^14^. The second option might revolve around the crystallization of surfactant in a semi-crystalline layer on the surface of the particles. Extremely dilute crystallites have also shown to produce large SFS intensities^15^. It is additionally known in aerosol literature that due to differences in evaporation rates, particles size, morphology and crystallinity can drastically change once they are released in the aerosol chamber^16–18^.

Summarizing, performing vibrational surface SFS from aerosols with a diameter centered near 40 nm and a density of 10^6^ particles/mL^1^ is at odds with understanding based upon nonlinear light scattering theory as well as state of the art experiments conducted by numerous labs, on (metal/plasmonic) particles, droplets, harmonophores, liposomes and other objects with vastly different compositions in both liquid and solid media. The nonlinear light scattering data and theory obtained since the 1980’s generally agrees well with one another, and in that sense shows the same type of confidence as one has with linear light scattering^19^. That the Article this contribution is arising from^1^ shows data that deviates by a factor of ~10^7^ from current state of the art data and understanding is therefore extremely puzzling to us.

## Supplementary information

Supplementary Information

## Data Availability

The data sets generated and analyzed during the current study are available from the corresponding author upon reasonable request.
