# Supplementary material for: A discrepancy of 107 in experimental and theoretical density detection limits of aerosol particles by surface nonlinear light scattering
Source: Commun Chem. 2023 Jun 8;6:114. doi: 10.1038/s42004-023-00903-8 (PMC10250540; doi:10.1038/s42004-023-00903-8)
Supplement: Supplementary file 1 — Supplementary Information [file 42004_2023_903_MOESM1_ESM.pdf]

Supplementary Information for:

A discrepancy of 10^7 in experimental and theoretical density detection limits of aerosol particles by surface nonlinear light scattering

by

Arianna Marchioro[1], Thaddeus W. Golbek[2], Adam S. Chatterley[2], Tobias Weidner[2], and Sylvie Roke[1]*

[1]Laboratory for Fundamental BioPhotonics (LBP), Institute of Bioengineering (IBI), and Institute of Materials Science (IMX), School of Engineering (STI), and Lausanne Centre for Ultrafast Science (LACUS), École Polytechnique Fédérale de Lausanne (EPFL), CH-1015 Lausanne, Switzerland.
[2]Department of Chemistry, Aarhus University, 8000 Aarhus C, Denmark; *e-mail: sylvie.roke@epfl.ch

Supplementary Methods

Sample preparation. All glassware was cleaned by thoroughly rinsing with ultrapure water (H_2O, Milli-Q UF Plus, Millipore, Inc., electrical resistance of 18.2 MΩ•cm) and ethanol (ethanol absolute, VWR). The emulsions were prepared according to a previous procedure.[1] Briefly, emulsions were prepared with a 2 % v/v of d_{34}-hexadecane oil (98%, Cambridge Isotope Laboratories Inc.) in deuterium oxide (D_2O, 99.90 % D, Eurisotope). The solutions were mixed with 8 mM sodium dodecyl sulfate (SDS, Sigma Aldrich) as the surfactant, using a hand-held homogenizer (TH, OMNI International) for 1 minute and an ultrasonic bath (35 kHz, 400 W, Bandelin) for 5 minutes. The size distribution of the nanodroplet sample was measured with dynamic light scattering (DLS, Malvern ZS Nanosizer) set to 90° collection angle with a wavelength of 633 nm.[2] The nanodroplets DLS data is plotted in the inset of Fig. 1C and the hydrodynamic diameter and polydispersity index (PDI) are 218 ± 2 nm and 0.251 ± 0.015, respectively. Hydrodynamic diameters are calculated from the intensity autocorrelation function using the optical properties of the liquids (d_{34}-hexadecane, D_2O). Samples were prepared freshly before each measurement. The samples were measured in sealed cuvettes (Hellma GmbH, 124-0.2-40, Germany) with a path length of 200 µm, with one side made of CaF_2 (22.0 mm x 1.0 mm, Crystran) and the other of quartz (Hellma GmbH, 124-0.2-40, Germany).

Sum Frequency Scattering (SFS) vibrational spectroscopy. The setup is based on a commercial 18 W, 190 femtosecond Yb-based laser/amplifier laser system (Pharos, Light Conversion) with pulses centered at 1032 nm and a repetition rate of 10 kHz. The output is used to generate tunable mid-IR pulses centered at 3450 nm with a dual channel optical parametric amplifier (ORPHEUS, Light Conversion) equipped with a difference frequency generator (LYRA,

Light Conversion). The generated mid-IR pulses are <100 fs with 13.6 µJ per pulse. Part of the output is used to pump a second-harmonic bandwidth compressor (SHBC, Light conversion) that generates a 2 ps visible pulse (FWHM 0.18 nm) centered at 515 nm with 7.5 µJ per pulse. The polarization of the visible beam is controlled by a Glan-Laser polarizer (GL10-A, Thorlabs) followed by a zero-order half-wave plate (WPH10M-514, Thorlabs). The power of the visible beam is controlled by a zero-order half-wave plate (WPH10M-514, Thorlabs) before the Glan-Laser polarizer. The mid-IR beam polarization is controlled by a low-order half-wave plate (WPLH05M-3500, Thorlabs) and cleaned-up with a BaF_2 wire-grid polarizer (WP25H-B, Thorlabs). The two beams, visible and mid-IR, are made collinear by reflecting the visible beam off a custom silver mirror with a 6 mm hole in the center drilled a 45° angle, while the mid-IR beam passes through the hole from the backside of the mirror. The two beams are focused onto the sample by a protected silver off-axis parabolic mirror (MPD129-P01, reflective focal length (RFL) = 50.8 mm, Thorlabs). The SFS light is collimated by an f = 50 mm plano-convex lens (LA1131-A, Thorlabs) at a scattering angle of 50°. The polarization of the SFS light is controlled by an achromatic half-wave plate (AHWP10M-580, Thorlabs) and polarizing beamsplitter cube (PBS251, Thorlabs). The beam is further filtered by two short-pass filters (FESH0500, Thorlabs) before being focused by a f = 40 mm plano-convex lens (LA1422-A, Thorlabs) into the slit and spectrally dispersed by a spectrograph (Kymera 328i, Andor) onto an iCCD camera (iStar 334, Andor). The polarization was set to PPP polarization (P-IR, P-visible, and P-SF) and the acquisition time was set to 180 s. The spectra were collected in the CH vibrational stretching region (2800 – 3000 cm^{-1}). Each dilution of the nanoemulsion was made with 8 mM SDS solution, so that the concentration of SDS was constant and only the particle density changed during the experiment. It is important to note that micelles, that are likely present since the SDS concentration is near the critical micelle concentration, have been shown to not generate enough signal to contribute to the spectral intensities measured for similar experiments.[3] The SFS spectra were normalized against a SFS spectra of a fine-grained sandpaper scratched quartz window. Each SFS sample spectra was collected at least 10 times (10 x 180 s) in order to be comparable to the 100 kHz experiments.[4]

Second Harmonic Scattering (SHS). The setup is based on a commercial 20 W, 190 femtosecond Yb-based laser/amplifier laser system (Pharos, Light Conversion) with pulses centered at 1032 nm and a repetition rate of 200 kHz. The incoming beam polarization is controlled by a Glan-Taylor polarizer (GT10-B, Thorlabs) and zero-order half wave plate (WPH10M-1030). The input beam, with pulse energies of 0.4 µJ, is focused by a f = 75 mm plano-convex lens (LA1257-B, Thorlabs) and filtered (FELH0850, Thorlabs) before entering a cylindrical glass sample cell (4.2 mm inner diameter, LS instruments). The scattered SH light is collimated

by a f = 50 mm plano-convex lens (LA1131-A, Thorlabs), polarized by a Glan-Taylor polarizer (GL10-A), filtered (FBH515-10), and focused with a f = 25.4 mm plano-convex lens (LA1252-A, Thorlabs) onto a photon counting head (H12386-210, Hamamatsu). The photon counting head is read out by a SR400 gated photon counter (gate = 5 ns, Stanford Research). Data points were collected at the scattering angle that corresponded to the highest intensity for the emulsion sample, which was 40° measured in air. An iris set to 10 mm diameter in front of the collection lens was used to control the acceptance angle. The sample was collected in PPP polarization, all beams in the horizontal plane (P). Dilutions of the prepared sample were measured from 0.2 % to 0.00002 % and measurement acquisition settings were set to 500 x 0.5s for each sample (Fig. 1D). The solution used to dilute the SDS nanoemulsion contained 0.8 mM SDS, so that the concentration of SDS was constant and only the particle density changed during the experiment. Error bars in each sample were calculated using standard error and propagation of error. The SHS data is normalized by subtracting the hyper-Rayleigh scattering (HRS) intensity of the solution containing SDS recorded in PPP polarization and dividing by the HRS intensity of D_2O (S1) recorded in the SSS polarization.[5]

$$S(\theta)_{PPP} = \frac{I(\theta=40°)_{PPP,\ SDS\,emulsion} - I(\theta=40°)_{PPP,\ bulk\,solution}}{I(\theta=40°)_{SSS,\ D_2O}} \qquad \text{(S1)}$$

Where I(θ) is the measured intensity. All data was collected at room temperature (23.3 $°C$).

Supplementary References

1. Chen, Y., Jena, K. C., Lütgebaucks, C., Okur, H. I. & Roke, S. Three Dimensional Nano "Langmuir Trough" for Lipid Studies. *Nano Lett.* 15, 5558–5563 (2015).

2. Jin, L., Jarand, C. W., Brader, M. L. & Reed, W. F. Angle-dependent effects in DLS measurements of polydisperse particles. *Meas. Sci. Technol.* 33, 045202 (2022).

3. Aguiar, H. B. de, Beer, A. G. F. de, Strader, M. L. & Roke, S. The Interfacial Tension of Nanoscopic Oil Droplets in Water Is Hardly Affected by SDS Surfactant. *J. Am. Chem. Soc.* 132, 2122–2123 (2010).

4. Qian, Y.; Brown, J. B.; Huang-Fu, Z.-C.; Zhang, T.; Wang, H.; Wang, S.; Dadap, J. I.; Rao, Y. In situ analysis of the bulk and surface chemical compositions of organic aerosol particles. *Comm. Chem.* 5, 58 (2022).

5. Smolentsev, N. & Roke, S. Self-Assembly at Water Nanodroplet Interfaces Quantified with Nonlinear Light Scattering. *Langmuir* 36, 9317–9322 (2020).
